# Treatment of Onychomycosis and the Drug–Drug Interactions in Patients with Diabetes Mellitus and Diabetic Foot Syndrome: A Systematic Review

**DOI:** 10.3390/idr17010004

**Published:** 2025-01-09

**Authors:** David Navarro-Pérez, Aroa Tardáguila-García, Sara García-Oreja, Francisco Javier Álvaro-Afonso, Mateo López-Moral, José Luis Lázaro-Martínez

**Affiliations:** 1 Diabetic Foot Unit, Clínica Universitaria de Podología, Facultad de Enfermería, Fisioterapia y Podología, Universidad Complutense de Madrid, Instituto de Investigación Sanitaria del Hospital Clínico San Carlos (IdISSC), 28040 Madrid, Spain; davinava@ucm.es (D.N.-P.); alvaro@ucm.es (F.J.Á.-A.); matlopez@ucm.es (M.L.-M.); diabetes@ucm.es (J.L.L.-M.); 2 Clínica Universitaria de Podología, Facultad de Enfermería, Fisioterapia y Podología, Universidad Complutense de Madrid, Instituto de Investigación Sanitaria del Hospital Clínico San Carlos (IdISSC), 28040 Madrid, Spain; sagarc14@ucm.es

**Keywords:** onychomycosis, diabetes, diabetic foot, drug interaction, antifungal

## Abstract

**Background:** This systematic review reports on treatments for onychomycosis in patients with diabetes and the drug interactions with other drugs in regard to the complicated diabetic patient profile. **Methods:** The recommendations in the preferred reporting items for systematic reviews and meta-analysis (PRISMA) checklist were applied and the included studies were evaluated using the Consolidated Standards of Reporting Trials (CONSORT) statement and the Strengthening the Reporting of Observational Studies in Epidemiology (STROBE) statement. Searches were conducted in November 2023, using the PubMed (Medline), Scopus, Cochrane Library, and Web of Science databases; studies on antifungal treatments for onychomycosis in patients with diabetes were included. Two authors performed the study selection and data extraction, and any discrepancies between the two reviewers were resolved through discussions with a third reviewer. This review was registered in PROSPERO (CRD42023442107). **Results:** The systematic review included 10 studies that met the selection criteria. Mycological cures for mild to moderate onychomycosis were: *Ageratina pichinchensis* (8.6%), 8% ciclopirox (8.6% 24 weeks and 54.3% 48 weeks), 10% efinaconazole (56.5–58.33%), terbinafine (73–76.6%), itraconazole (88.2%), and laser therapy (43.8%). No serious adverse effects or drug interactions were observed because patients with major complications, such as peripheral vascular disease, diabetic neuropathy, liver and renal dysfunction, poorly controlled diabetes, and severe onychomycosis, were excluded. **Conclusions:** The antifungal treatments described in the included studies are safe for patients with well-controlled diabetes, but there are currently no studies involving patients with diabetes and multiple complications, such as diabetic foot syndrome or severe onychomycosis. Thus, further research is needed in terms of this patient profile.

## 1. Background

Diabetes causes a sustained increase in blood glucose levels and can lead to complications such as retinopathy, nephropathy, and diabetic neuropathy [1]. Diabetes affects 537 million people worldwide [1]. It is estimated that around 25% of these patients will develop a diabetic foot ulcer in their lifetime, with increased morbidity, disability, health and social care costs, and mortality [2].

Diabetic foot syndrome is defined as the infection, ulceration, or destruction of the tissues of the foot of a person with previously diagnosed diabetes mellitus. Neuropathy and/or peripheral arterial disease of the lower limbs [2] are risk factors for the development of diabetic foot ulcers [3,4,5]. Foot ulcers are found in 12–25% of people with diabetes and precede 84% of all non-traumatic amputations [6].

Chronically maintained hyperglycemia, neuropathy, and peripheral arterial disease lead to the deterioration of the immune system, by affecting polymorphonuclear leukocytes and phagocytic functions [7]. These conditions are common in people with diabetes and especially those with diabetic foot syndrome. Thus, there is an increased risk of fungal and bacterial skin infections, as well as fungal nail infections [8,9].

Indeed, people with diabetes have a three-fold higher risk of developing onychomycosis (ONM) than people without diabetes [6,8]; up to one-third of the diabetic population [10] has developed onychomycosis, as shown in a systematic review previously published by Navarro-Pérez et al. (2023) [11].

Onychomycosis is a fungal infection of the nails that affects approximately 5.5% of the world’s population and accounts for half of all nail infections [12]. It is characterized by nail thickening, subungual hyperkeratosis, discoloration, and onycholysis, among others. The prevalence of the disease increases with the following risk factors: advanced age, male gender, abnormal nail morphology, diabetes mellitus, genetic factors, wearing certain types of footwear, peripheral arterial disease, and immunodeficiency [13,14,15].

Onychomycosis has several clinical presentations: distal and lateral subungual onychomycosis (DLSO), proximal subungual onychomycosis, superficial white onychomycosis, and total dystrophic onychomycosis [12]. DLSO is the most common form of onychomycosis and often results from tinea pedis. Total dystrophic onychomycosis is the most severe form [16] and can cause severe lesions. Nail thickening and subungual detritus can lead to subungual ulcers, due to the pressure on the nail plate leading to a diabetic foot ulcer [6]. Onychomycosis can also be classified according to nail involvement, using indices such as the Onychomycosis Severity Index [17,18].

Therapeutic options include topical treatments applied directly to the affected area, oral treatments, essentials oils, and even external therapies, such as laser, iontophoresis, or photodynamic therapy [16,19]. In some cases, combined treatment may even be necessary and indicated depending on the location, area of involvement, and the causative agent [20], as well as a previous diagnosis through the use of microbiological culture, the polymerase chain reaction (PCR), direct microscopy (e.g., KOH), or histological techniques [12,16,21]. Therefore, to prevent diabetic foot ulcers and the downstream effects, there is an urgent need for the treatment of onychomycosis in patients with diabetes.

In severe cases of onychomycosis, there may be subungual lesions and, thus, the use of oral therapies is recommended. Problems emerge when analyzing the possible interactions of the drugs taken by this patient profile with oral antifungal treatments.

Therefore, we noticed a need for this systematic review, to compile and analyze the results of the antifungal treatments available for the treatment of onychomycosis in patients with diabetes and diabetic foot syndrome. This review also describes drug interactions with other medications.

## 2. Methods

The preferred reporting items for systematic reviews and meta-analyses (PRISMA) checklist was used for this systematic review [22]. The included studies were evaluated using the Consolidated Standards of Reporting Trials (CONSORT) statement and the Strengthening the Reporting of Observational Studies in Epidemiology (STROBE) statement [23,24]. This systematic review was registered in PROSPERO (an international prospective register of systematic reviews; identification code CRD42023442107).

### 2.1. Literature Search

All the searches were conducted in November 2023. The PubMed (Medline), Scopus, Cochrane Library, and Web of Science databases were searched to identify studies evaluating treatments for onychomycosis in patients with diabetes mellitus.

The electronic database search was performed using the keywords ‘onychomycosis’ and ‘diabetes’; ‘Terbinafine’ and ‘Diabetes’; ‘Itraconazole’ and ‘Diabetes’; and ‘Ciclopirox’ and ‘Diabetes’. Studies published in English, Spanish, French, and German were evaluated.

The titles and abstracts were reviewed to exclude studies that did not meet the selection criteria. The full text of the article was subsequently analyzed to determine whether the study met all the inclusion criteria and none of the exclusion criteria.

### 2.2. Article Selection

The articles were selected based on the inclusion and exclusion criteria, which are described below.

The inclusion criteria were as follows: clinical studies, clinical trials, comparative studies, controlled clinical trials, guidelines, multicenter studies, observational studies, and randomized controlled trials, concerning the treatment of onychomycosis in patients with diabetes mellitus. The participants were men or women of any age and the studies were published in English, French, German, or Spanish, without a publication date limit.

The exclusion criteria were animal studies; pre-clinical or in vitro studies; and studies with insufficient data for analysis.

The references from the studies were also examined to identify additional articles.

The search and article selection were performed by two independent reviewers (D.N.P and A.T.G). Any discrepancies between the two reviewers were resolved through discussions with a third reviewer (J.L.L.M).

### 2.3. Data Extraction

A customized Microsoft Excel spreadsheet (Excel 2016, Microsoft, Redmond, WA, USA) was used to extract the data from the studies. The extracted data included the authors’ names, publication year, study design, intervention, number of participants, cure rate, follow-up, diagnostic test used, etiological agent, number of treatments required, adverse events, and relevant data.

### 2.4. STROBE and CONSORT Guidelines

The CONSORT and STROBE guidelines were developed to help guarantee the high quality presentation of reporting of trials and observational studies [23,24]. The reviewers evaluated the adequacy of the reported items using both guideline checklists. These checklists provide a framework to ensure completeness and transparency. There are 22 items in the STROBE guideline checklist, including the following: item 1, title and abstract; items 2 and 3, introduction; items 4 to 12, methods; items 13 to 17, results; items 18 to 21, discussion; and item 22, funding and sponsorship. There are 25 items in the CONSORT guideline checklist, including the following: item 1, title and abstract; item 2, introduction; items 3 to 12, methods; items 13 to 19, results; items 20 to 22, discussion; and items 23 to 25, other information.

Two reviewers (D.N.-P. and A.T.-G.) independently assessed each study using the CONSORT and STROBE guidelines. A third reviewer (J.L.L.-M.) helped to achieve consensus in case of disagreement.

### 2.5. Levels of Evidence and Grades of Recommendation

The type of study was also recorded and classified according to the levels of evidence and grades of recommendation proposed by the Oxford Centre for Evidence-Based Medicine (OCEBM) [25]. The quartile in relation to the Science Citation Index Expanded (SCIE) and impact level of the journals in which the articles were published, were also collected.

### 2.6. Outcome Measures

Information about the patient demographics, study sample size, and the participant/treatment group was extracted. The main measure in this systematic review was the treatments for onychomycosis in patients with diabetes described in the literature, the cure rate of these treatments (mycological and complete cure), and their interaction with other drugs.

The definition of clinical cure varies between studies, from 0% nail plate involvement to >50% clinical improvement [12]. A mycological cure is a negative result in microbiological tests and a complete cure is a clinical cure, accompanied by a negative laboratory test result [12].

## 3. Results

### 3.1. Quality of the Reporting

Items 9 (bias), 10 (study size), 11 (quantitative variables), and 16 (main results) were the most poorly completed in the included studies. Table 1 shows the overall rating for the STROBE checklist. Items 7 (study size), 8–10 (randomization), 20 (limitations), 23 (register number), and 24 (protocol) were the most poorly completed in regard to the included studies. Table 2 shows the overall rating for the CONSORT checklist.

### 3.2. Literature Search

**Figure 1:**
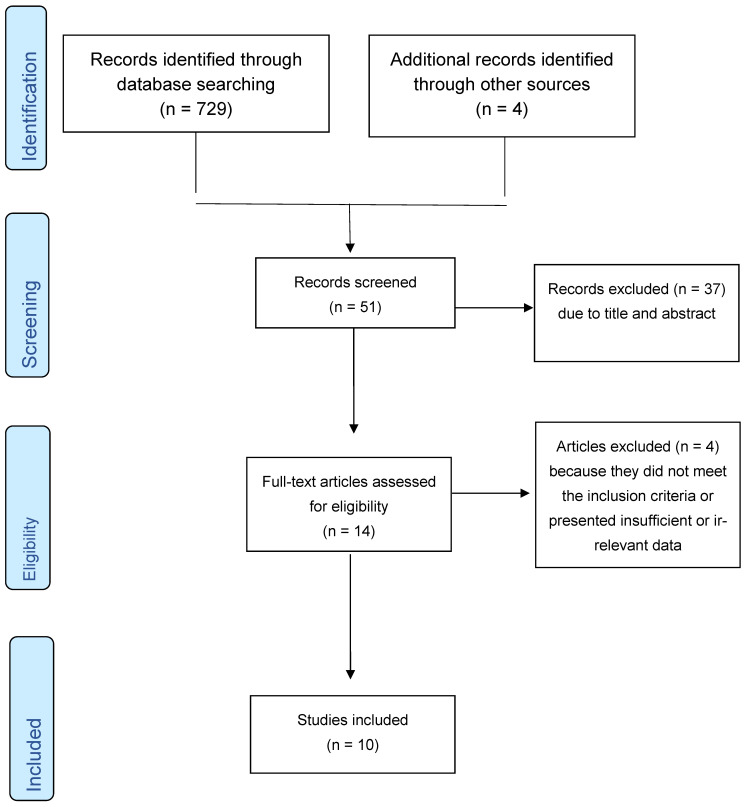
Flowchart for the identified studies.

A total of 733 results were identified as a result of the initial search strategies. After applying the selection criteria, the search result was refined to 63. Eliminating repeated results gave us a total of 51 studies. Reading the title and abstract led to the exclusion of 37 articles. Four articles were excluded after reading the remaining 14 articles, resulting in a total of 10 articles in this systematic review (Figure 1). The data extracted from the studies included in this systematic review are summarized in Table 3.

### 3.3. Study Characteristics

The 10 included studies [26,27,28,29,30,31,32,33,34,35] were published between 1997 and 2020. Six studies were RCTs (evidence level 1b and grade A recommendation) [30,31,32,33,34,35] and four were case series (evidence level 4 and grade C recommendation) [26,27,28,29] (Table 4).

Seven of the articles provide data on the cure rate of the treatments, while three focus exclusively on safety and adverse events.

The treatments addressed in the articles are: *Ageratina pichinchensis*, 8% ciclopirox nail lacquer topical solution, oral pulse itraconazole, oral continue terbinafine, milling of the nail, Nd-YAG 1064 nM laser, 10% efinaconazole placebo, and sham treatment.

### 3.4. Diagnostic Test and Etiological Agent

All the studies diagnosed onychomycosis using the KOH test and a mycological culture, except for one article that carried out a diagnosis on a clinical basis only, without additional tests.

However, only five of the nine studies that performed diagnostic tests specified the pathogens causing the onychomycosis [27,28,30,31,34]. In all of them, the most frequent pathogen was *Trichophyton rubrum,* followed by *Trichophyton mentagrophytes*.

### 3.5. Treatment Effectiveness

In the study by Romero-Cerecero et al. [30], *Ageratina pichinchensis* was presented as an alternative to 8% ciclopirox, showing a mycological cure rate of 7.1% and 8.6%, respectively, after once daily application for 24 weeks. This work concluded that these therapies are effective treatments for mild and moderate onychomycosis in patients with type 2 diabetes. Brenner et al. [26] agree that 8% ciclopirox is an effective treatment, as they obtained a mycological cure rate of 54.3%, when applied once daily for 48 weeks, in patients with type 2 diabetes.

The 10% efinaconazole treatment was also discussed as a topical treatment. Vlahovic et al. [35] compared the results of 10% efinaconazole against a placebo, obtaining a mycological cure rate of 56.5%, after once daily application for 52 weeks. Shofler et al. [28] obtained similar results, with a cure rate of 58.33%, after once daily application for 50 weeks.

In terms of oral therapy, continuous terbinafine (250 mg once daily for 12 weeks) had a mycological cure of 76.6% in the work by Gupta et al. [31] and 73% in the study by Farkas et al. [27]. A pulsatile itraconazole regimen (200 mg twice daily during the first week of each month for three months) showed a mycological cure rate of 88.2% in the work by Gupta et al. [31].

Finally, the laser therapy described in the study by Nijenhuis-Rosien et al. [34] led to a mycological cure rate of 43.8%, after four applications, versus 41.9% in the control group, in which only the nails were milled during a follow-up after one year, and they concluded that there was still not enough evidence to determine whether the treatment was effective, despite it being a treatment that was on the increase.

### 3.6. Adverse Events

Adverse events reported after the application of topical treatments included periungual skin irritation and the appearance of small vesicles that were always resolved within seven days. Both itraconazole and terbinafine have been shown to be safe treatments in patients with diabetes, with isolated cases of gastrointestinal problems, elevated liver function scores, skin rashes, headaches, and respiratory problems. No drug–drug interactions were observed in regard to the patients included in the studies. There were no alterations in the patients’ blood glucose or glycosylated hemoglobin values, making them safe and effective therapies.

During the palliation in terms of the laser therapy, 9.4% of the patients experienced pain, with no other adverse events beyond the sensation of pain reported.

### 3.7. Inclusion and Exclusion Criteria Described in the Studies

The inclusion and exclusion criteria used in the included studies can be found in Table 5.

## 4. Discussion

To the best of our knowledge, this is the first systematic review in the last 15 years to evaluate onychomycosis treatments in patients with diabetes, as well as the drug–drug interactions or adverse events that may occur.

The heterogeneity of the study design, the variety of treatments evaluated, the follow-up in each of them, and the profile of the patients included, makes the overall analysis of the effectiveness of the treatments inconsistent. Therefore, we must look at the profile of the patients included in the study to better understand the effectiveness and adverse events unique to each of the treatments.

Most of the included studies had no severe adverse effects specific to diabetes drug interactions. However, this may be mostly due to the exclusion criteria applied in the studies (Table 5). Eight of the ten studies [26,28,29,30,31,32,34,35] excluded patients with major complications, such as peripheral vascular disease, diabetic neuropathy, liver dysfunction, a lack of hepatic and/or renal control, immunosuppressant treatment, ischemic pain, renal failure, uncontrolled diabetes, corticosteroid use, nephropathy, blood disorders, and severe onychomycosis. Patients with diabetic foot syndrome have many of these characteristics, making it difficult to assess which antifungal treatment to use in patients with complicated diabetes and, even more so, when these patients have diabetic foot syndrome.

Patients with diabetes are often multi-pathological patients and, thus, are polymedicated, i.e., they are receiving multiple systemic medications [36,37]. Thus, they are not good candidates in regard to disease management with oral antifungals. In particular, potential drug interactions between oral antifungals and oral hypoglycemic agents should be considered. For example, itraconazole is a potent inhibitor of cytochrome P-450 3A4 and may increase the concentration of drugs metabolized by this pathway [31]. In addition, cytochrome P-450 3A4 inhibitors or inducers may increase or decrease itraconazole concentrations, respectively [31]. Contraindicated drugs include immunosuppressants, calcium channel blockers, protease inhibitors, anti-arrhythmics, and, most importantly for patients with diabetes, oral hypoglycemic drugs [38]. One of the most widely used oral antidiabetic drugs (OADs), metformin, has no drug–drug interactions, due to its renal metabolism, rather than the hepatic metabolism involving other OADs. However, cases of severe hypoglycemia have been reported in patients co-administered with azole antifungals and oral hypoglycemic drugs [39,40].

Most drugs used in dermatology, including systemic antifungals, are metabolized in the liver, and the CYP 3A4 isoform is the most prevalent cytochrome isoform, accounting for 60% and 70% of hepatic and enterocyte cytochrome enzymes, respectively. In regard to itraconazole, the basis for some of the drug interactions is the inhibition/induction of the CYP 450 3A4 isoform [38,40]. For example, itraconazole will interact with the following medications:

- Benzodiazepines (midazolam, triazolam, alprazolam);
- Calcium antagonists of the dihydropyridine class (amlodipine, felodipine, nifedipine, etc.), leading to oedema of the ankles and legs due to high levels of calcium blockers;
- Cyclosporine may increase in concentration and nephrotoxicity may occur;
- Hypocholesterolemic agents (lovastatin, simvastatin, atorvastatin, cerivastatin, etc.) and can lead to high plasma concentrations, which may be associated with rhabdomyolysis;
- Hypoglycemic agents (some sulfonylureas and non-sulfonylureas), with severe hypoglycemia having been reported in patients taking hypoglycemic agents and azoles simultaneously. Itraconazole should, therefore, either not be administered or very rigorous blood glucose monitoring should be maintained.

In contrast to other oral antifungals, terbinafine is an allylamine with a weak substrate interaction with cytochrome P-450; thus, it is rapidly metabolized and its interaction with other drugs is usually negligible. It inhibits fungal squalene epoxidase, without affecting the enzymatic activity of cytochrome P-450 3A. It has no inhibitory effects on this metabolic pathway and, therefore, has very few pharmacological effects [38]. However, caution should be exercised in regard to patients taking cyclosporine, rifampicin, and terfenadine. In addition, studies have been published that describe the interaction of terbinafine with some anticoagulants, such as warfarin; thus, this patient profile should be monitored closely [41,42]. Studies have recently been published in which the aspartate aminotransferase and alanine aminotransferase levels were higher in those taking terbinafine than in those taking itraconazole. One case involved liver damage after treatment with terbinafine. Thus, it is advisable to maintain strict control of this treatment in patients with diabetes being treated with oral antifungals [43,44].

An additional problem is the resistance currently being recorded in regard to the treatment involving dermatophytes with terbinafine, either for clinical reasons specific to the patient (such as diabetes) or for microbiological reasons, such as acquired resistance due to previous exposure to the drug. In other words, failure can be due to empirical prescription without diagnostic confirmation using a complementary test in patients who did not have onychomycosis before or who had been impacted by another pathogen [45,46,47]. Thus, it is important to confirm the diagnosis via a complementary test before prescribing appropriate antifungal drugs [21,46].

Interactions by antifungal drugs involving some of these complications have been reported, but studies that include close follow-up in terms of this patient profile are needed, because the failure to treat onychomycosis in patients with diabetic foot syndrome may lead to subungual ulcers [48]. In general, mild onychomycosis is not a major problem, but more severe and untreated onychomycosis can be a problem for patients with neuropathy and peripheral vascular disease, because thickened and splitting nails can increase pressure on the nail plate and injure the surrounding skin [48,49]. The neuropathic patient does not feel these small lesions, but they become an entry point for bacteria, which can cause infections, such as paronychia and cellulitis. In turn, this can endanger the affected fingers or even the limb, depending on the severity of the infection [48,49]. Special care must be taken in regard to subungual lesions, because the subungual bed and the phalanges are very close to each other, and this can lead to bone infections, thus retarding the healing of the ulcer [32,48,49,50,51,52]. Therefore, the treatment of onychomycosis combined with nail debriding is recommended, especially in the presence of dermatophytoma, where the density of the biofilm-like fungal mass may reduce the effect of the antifungal agent; patient education is also needed [48,49,53].

Another exclusion criteria applied in the included studies that biases the validity of the results is the presence of severe onychomycosis or onychodystrophic onychomycosis. Patients with diabetic foot syndrome are often older or have vascular complications, thus they tend to present with onychogryphosis of the nails [54,55,56,57].

Therefore, the exclusion criteria applied in the included studies make it difficult to generalize the results to the whole population with diabetes, because only patients with well-controlled diabetes or without severe complications are included.

Another interesting fact is that when these studies were published it was estimated that the global population with diabetes would be 300 million in 2025 or 366 million by 2030 [31,32,58], but today, in 2024, 537 million people have diabetes [2]. Thus, the estimates made at that time in regard to onychomycosis in patients with diabetes may now be much higher than what was previously estimated.

Foley et al. [16] conducted a Cochrane Library review in 2020, describing the effectiveness of topical treatments, and concluded that although studies support this type of treatment, the mycological and complete cure rates are relatively low. They further concluded that alternative treatments, such as laser therapy, have not acquired sufficient evidence and might cause subungual lesions if applied at high potencies.

A recent study described 8% ciclopirox with SLS, beta-cyclodextrins, and poloxamer-407 (Ciclo-Tech^®^ technology, Barcelona, Spain). These vehicles can penetrate more easily into the nail plate and increase the antifungal effect of ciclopirox [59]. However, there were no statistically significant differences compared to the control group. Although the study only included patients with mild and moderate onychomycosis, this strategy could be a good alternative, and it would be interesting to perform new studies in the future, applying topical therapies and a thorough debridement of the nail plate, in combination with other therapies that do not cause lesions or drug interactions in patients with poorly controlled diabetes; neuropathy; peripheral vascular disease; diabetic foot; and moderate, severe, or dystrophic onychomycosis. These issues can have the greatest consequences. Some alternative therapies currently under investigation include the use of essential oils (EOs), such as *Ageratina pichinchensis*, *Thymus vulgaris*, *Cinnamomum zeylanicum,* or *Melaleuca alternifolia*. EOs may offer significant advantages, particularly in patients with diabetes mellitus, as they do not cause drug–drug interactions or hepatotoxicity. However, they should be further studied as some cases of allergies and skin irritation have been reported [60,61,62,63,64].

This systematic review does have some limitations. All the studies have several negative items in regard to the STROBE and CONSORT guidelines. There was heterogeneity in terms of the study design and the diversity of the treatments impeded meta-analysis. The inclusion of articles written only in English, Spanish, French, or German is another limitation.

## 5. Conclusions

The antifungal treatments described here are safe in patients with well-controlled diabetes, but there are currently no studies on patients with diabetes and multiple complications, such as diabetic foot syndrome or severe onychomycosis.

Healthcare professionals should make a correct diagnosis by specifying the causative organism through the use of valid complementary tests, before prescribing an oral antifungal drug in order to avoid creating resistance in patients. They should consider the medications and comorbidities that patients may have, such as renal or hepatic insufficiency, the administration of drugs whose interactions are doubtful, or the administration of cytochrome P-450 inhibitor drugs. They should closely monitor these patients during the application of the treatment.

The studies described in the literature focus on patients with well-controlled diabetes; thus, new studies are needed in regard to the profile of patients with poorly controlled diabetes, diabetic foot syndrome, or severe onychomycosis, thus creating alternatives for these patients, and new research directions may include emerging therapies, such as essential oils, which offer significant advantages, as they do not cause drug interactions or hepatotoxicity.

## Figures and Tables

**Table 1:** Overall rating for Strengthening the Reporting of Observational Studies in Epidemiology (STROBE).

Item Number from STROBE Guidelines																							
	1 Title	1 Abstract	2	3	4	5	6	7	8	9	10	11	12	13	14	15	16	17	18	19	20	21	22
Brenner MA (2007) [26]	Yes	Yes	Yes	Yes	Yes	Yes	Yes	Yes	Yes	No	Yes	No	Yes	Yes	Yes	Yes	No	Yes	Yes	No	Yes	No	Yes
Farkas B (2002) [27]	Yes	Yes	Yes	Yes	Yes	Yes	Yes	Yes	Yes	No	No	Yes	Yes	Yes	Yes	Yes	Yes	Yes	Yes	No	Yes	Yes	Yes
Shofler D (2020) [28]	No	Yes	Yes	Yes	Yes	No	Yes	Yes	Yes	No	No	No	No	Yes	Yes	Yes	No	No	Yes	Yes	Yes	Yes	Yes
Pollak R (1997) [29]	No	Yes	Yes	Yes	Yes	Yes	Yes	Yes	No	No	No	No	No	Yes	Yes	Yes	No	No	Yes	No	Yes	Yes	No

Green color: the study included the completion of the item. Red color: the study did not include the completion of the item.

**Table 2:** Overall rating for Consolidated Standards of Reporting Trials (CONSORT).

Item Number from CONSORT Guidelines																										
	1 Title	1 Abstract	2	3	4	5	6	7	8	9	10	11	12	13	14	15	16	17	18	19	20	21	22	23	24	25
Romero-Cerecero O (2020) [30]	No	Yes	Yes	Yes	Yes	Yes	Yes	No	No	No	No	Yes	Yes	Yes	Yes	Yes	Yes	Yes	Yes	Yes	No	Yes	Yes	Yes	No	No
Gupta AK (2006) [31]	No	Yes	Yes	Yes	Yes	Yes	Yes	No	Yes	Yes	Yes	Yes	Yes	Yes	Yes	Yes	Yes	Yes	Yes	Yes	No	Yes	Yes	No	No	No
Albreski DA (1999) [32]	No	Yes	Yes	No	Yes	No	Yes	No	No	No	No	No	Yes	Yes	Yes	Yes	Yes	No	No	Yes	No	Yes	Yes	No	No	Yes
Armstrong DG (2005) [33]	Yes	Yes	Yes	Yes	Yes	Yes	Yes	No	No	No	No	No	Yes	No	No	Yes	Yes	Yes	Yes	Yes	No	Yes	No	No	No	Yes
Nijenhuis-Rosien L (2019) [34]	Yes	Yes	Yes	Yes	Yes	Yes	Yes	Yes	Yes	Yes	Yes	Yes	Yes	Yes	Yes	Yes	Yes	Yes	Yes	Yes	Yes	Yes	Yes	Yes	Yes	Yes
Vlahovic TC (2014) [35]	No	Yes	Yes	Yes	Yes	Yes	Yes	No	No	No	No	No	Yes	Yes	No	Yes	Yes	Yes	Yes	Yes	Yes	Yes	Yes	No	No	Yes

Green color: the study included the completion of the item. Red color: the study did not include the completion of the item.

**Table 3:** Characteristics of studies included in the systematic review. Abbreviations: DM2, diabetes mellitus type 1; KOH, potassium hydroxide; RCT, randomized controlled trial; *T. mentagrophytes*, *Trichophyton mentagrophytes*; *T. rubrum*, *Trichophyton rubrum*; NA, not applicable; NID, non-insulin dependent; and ID, insulin dependent.

First Author (Year)	Type of Study	Intervention	Number of Participants	Cure Rate	Follow-Up/Evaluation Period	Etiological Agent		Number of Treatments Required	Adverse Events	Relevance/Interesting Facts
						Diagnostic Test	Micro-Organism			
Romero-Cerecero O (2020) [30]	RCT	Arm 1: Ageratina pichinchensis Arm 2: Ciclopirox 8% nail lacquer topical solution	Arm 1: 35 Arm 2: 36	Arm 1: 7.1% Arm 2: 8.6% (complete cure)	24 weeks	KOH and mycological culture	*T. rubrum* (32.4%) *T. mentagrophytes* (28.2%) *Epidermophyton floccosum* (14.1%) *Candida* spp. (25.3%)	Once a day, every third day, during the first eight weeks. Twice a week until the end of 24 weeks.	Irritation of the skin surrounding the nail (less than 7 days).	Clinical efficacy 77.2% (control group) and 78.5% (experimental group) in the treatment of mild and moderate onychomycosis. No statistically significant difference.
Gupta AK (2006) [31]	RCT	Arm 1: Oral pulse itraconazole Arm 2: Oral continuous terbinafine	Arm 1: 34 Arm 2: 29	Arm 1: 88.2% Arm 2: 76.7% (mycological cure)	48 weeks	KOH and mycological culture	*T. rubrum* (80%) *T. mentagrophytes* (15.7%) *Epidermophyton floccosum* (4.3%)	Itraconazole 200 mg twice daily during the first week of each month for three consecutive months. Terbinafine 250 mg once daily for 12 weeks.	Gastrointestinal problems.	Both treatments were safe and there were no interactions with concomitant medications. DLSO dermatophyte. No statistically significant difference.
Albreski DA (1999) [32]	RCT	Arm 1: Oral pulse itraconazole 2v/d Arm 2: Standard palliative care	Arm 1: 27 Arm 2: 25	Arm 1: NA Arm 2: NA	3 months	KOH and mycological culture	NA	Itraconazole 200 mg twice daily during the first week of each month for three consecutive months.	Elevated liver function test results, skin eruption, diarrhea, and pedicle edema.	Itraconazole therapy was found to be safe for the treatment of distal dermatophytic subungual onychomycosis. Hemoglobin A1c values were higher, but this was not statistically significant (*p* > 0.05). No statistically significant difference.
Armstrong DG (2005) [33]	RCT	Arm 1: Ciclopirox 8% nail lacquer topical solution Arm 2: None	Arm 1: 34 Arm 2: 36	Arm 1: NA Arm 2: NA	48 weeks	Clinical diagnosis	NA	Daily examination and application.	5.9% ulcerated at 48 weeks; 5.6% ulcerated at 48 weeks.	There may be no immediate prophylactic benefit from the use of a nail lacquer to prevent wounds in patients with foot risk category 2 and 3. No statistically significant difference.
Nijenhuis-Rosien L (2019) [34]	RCT	Arm 1: Nd-YAG 1064nM laser Arm 2: Sham treatment	Arm 1: 32 Arm 2: 31	Arm 1: 43.8% Arm 2: 41.9% (mycological cure)	52 weeks	KOH, mycological culture, or PCR	*T. rubrum* (74.6%) *T. mentagrophytes* (20.6%) *Epidermophyton floccosum* (1.6%) Other (3.2%)	Four applications.	Pain during the treatment (9.4% in the laser group and 12.9% in the sham group).	Treatment with a laser is safe, but is not effective. No statistically significant difference.
Vlahovic TC (2014) [35]	RCT	Arm 1: Efinaconazole 10% topical solution Arm 2: Vehicle (placebo)	Arm 1: 82 Arm 2: 30	Arm 1: 56.5% Arm 2: 14.8% (mycological cure)	52 weeks	KOH and mycological culture	NA	Once daily application.	Local site reactions.	Efinaconazole 10% topical solution is safe and effective. No statistically significant difference.
Brenner MA (2007) [26]	Case series	Ciclopirox 8% nail lacquer topical solution	49 (DM2) (16 insulin and 40 oral hypoglycemic)	54.3% mycological cure (both tests were negative)	48 weeks (8, 16, 24, 32, 40, and 48)	KOH and mycological culture	NA	Once daily for 48 weeks.	Nail disorder (6) and fungal infection (5).	85.7% mycological outcome related improvement or cure; ciclopirox was safe; includes only distal subungual onychomycosis.
Farkas B (2022) [27]	Case series	Oral terbinafine	89 (52 NID and 37 ID)	73% (71.1% NID and 75.7% ID)	48 weeks	KOH and mycological culture	*T. rubrum* (67.4%) *T. mentagrophytes* (19.1%) *Epidermophyton floccosum* (1.1%) Other (12.2%)	Terbinafine 250 mg once daily for 12 weeks.	Seven patients with ID (headache, 2; a temporary disturbance or loss of taste, 3; stomachache, 1; gastrointestinal disturbance, 1).	Practically unchanged for creatinine, aspartate aminotransferase, and for hematological parameters; 83% had the same blood glucose level after the 12-week treatment.
Shofler D (2020) [28]	Case series	Efinaconazole 10% topical solution	36	58.33% (KOH and culture negative)	50 weeks	KOH and mycological culture	*T. rubrum* (NA) *T. mentagrophytes* (NA)	Once daily for 50 weeks.	Vesicles appearing on the target toenail (2), cellulitis limited to the leg (1), and new-onset verruca (1).	Safe and efficacious. Not found to be associated with the level of glycemic control.
Pollak R (1997) [29]	Case Series	Oral terbinafine	77	NA	72 weeks	KOH and mycological culture	NA	Terbinafine 250 mg once daily for 12–24 weeks.	47 (61,01%) patients had skin, gastrointestinal, and respiratory problems.	No serious adverse events due to the oral terbinafine.

**Table 4:** Level of evidence, grade recommendation, Science Citation Index Expanded (SCIE) quartile, and impact factor.

Author (Year)	Level of Evidence	Recommendation Grade	SCIE Quartile	Impact Factor
Romero-Cerecero O (2020) [30]	1b	A	Q1	7.2
Gupta AK (2006) [31]	1b	A	Q1	9.2
Albreski DA (1999) [32]	1b	A	Q4	0.7
Armstrong DA (2005) [33]	1b	A	Q2	3.1
Nijenhuis-Rosien L (2019) [34]	1b	A	Q1	9.228
Vlahovic TC (2014) [35]	1b	A	Q4	1.5
Brenner MA (2007) [26]	4	C	Q4	0.7
Farkas B (2002) [27]	4	C	Q1	10.3
Shofler D (2020) [28]	4	C	Q4	0.6
Pollak R (1997) [29]	4	C	Q4	0.7

**Table 5:** Inclusion and exclusion criteria described in the studies. Abbreviations: DM, diabetes mellitus.

Author (Year)	Inclusion Criteria	Exclusion Criteria
Romero-Cerecero O (2020) [30]	Mild or moderate onychomycosis DM2 Last blood glucose rate < 180 mg/dL Disease evolution over 10 years	Severe onychomycosis Patients with onycholysis, diabetic foot syndrome, peripheral vascular insufficiency, and diabetic neuropathy
Gupta AK (2006) [31]	DM1 or DM2 Dermatophyte infection of at least one great toenail, with 10% target nail involvement	Baseline liver function tests elevated to more than twice the upper limit of a normal finding History of uncontrolled renal or hepatic disease Treatment with immunosuppressant drugs within 6 months
Albreski DA (1999) [32]	DM Distal dermatophytic subungual onychomycosis of the toenail	Presence of a serious concurrent medical condition History of psoriasis or hypersensitivity to imidazole or azole compounds Baseline liver function test results more than twice the upper limit of a normal finding Patients requiring lipid-lowering agents and warfarin Patients requiring rifampin, rifabutin, H2-blocking or proton-inhibiting agents, antacids used continually, phenobarbital, phenytoin, carbamazepine, astemizole, terfenadine, digoxin, midazolam, triazolam, cisapride, or HMG-CoA reductase inhibitors metabolized by the cytochrome P-450 3A enzyme system
Armstrong DG (2005) [33]	Foot risk category 2 (neuropathy/deformity) or category 3 (history of ulceration or amputation)	Patients unable to ambulate without the assistance of a wheelchair or crutches and, if they were sight impaired, to the extent that they were legally blind
Nijenhuis-Rosien L (2019) [34]	DM1 or DM2 Had risk factors for developing foot ulcers (defined as Sims classification 1 or 2)	Sims classification 0 or 3, ischemic rest pain in a leg Ankle–brachial index < 0.9, a toe pressure < 50 mmHg, having received renal replacement therapy or experiencing severe renal insufficiency, use of immunosuppressive drugs, the presence of psoriasis, lichen planus, or other abnormalities potentially involving the toenails
Vlahovic TC (2014) [35]	Those patients whose diabetes was controlled by diet or medication	Patients with uncontrolled diabetes (as determined by the investigator)
Brenner MA (2007) [26]	DM2 currently well-controlled, with medical intervention (insulin injection or oral agents) Diagnosis of distal subungual onychomycosis of at least one great toenail (target nail) Patients with medical visits regularly scheduled, in good general health, and with a good pulse in both feet	Serious diabetic foot conditions (for example, open wounds and surgery), severe plantar or moccasin tinea pedis A history of immunosuppression, overt signs of foot neuropathy, or known or suspected human immunodeficiency virus infection Patients who received systemic retinoids, immunosuppressive drugs
Farkas B (2002) [27]	Caucasian diabetic treated with oral antidiabetic agents or insulin Distal subungual onychomycosis of the toenails	Alcohol or drug abuse, planned or established pregnancy, lactation, inadequate contraception and sensitivity to the study medication
Shofler D (2020) [28]	Trichophyton rubrum or trichophyton mentagrophytes Involvement of a minimum 20% of the target great toenail was required for inclusion	Non-dermatophyte fungus infection, diagnosis of superficial white onychomycosis Peripheral arterial disease or anatomic abnormalities of the target toenail Systemic corticosteroid or immunomodulatory active interdigital tinea pedis
Pollak R (1997) [29]	Dermatophyte-caused onychomycosis of the toenails	Psoriasis, mucocutaneous candidiasis, or known immunodeficiencies Had liver disease, nephropathy, or blood disorders Had any disease that could significantly impair the gastrointestinal absorption of the drug Had baseline hepatic enzyme test results greater than 1.5 times the upper limit of normalcy Had a history of alcohol or substance abuse

## Data Availability

The data that support the findings in this study are available from the corresponding author upon reasonable request.
